# Synthesis and biological activity of 2-[6-methyl-4-(thietan-3-yloxy)pyrimidin-2-ylthio]acetohydrazide derivatives

**DOI:** 10.5599/admet.941

**Published:** 2021-02-18

**Authors:** Svetlana Meshcheryakova, Alina Shumadalova, Ozal Beylerli, Ilgiz Gareev

**Affiliations:** Bashkir State Medical University, Ufa, Russia

**Keywords:** thietan, thiopyrimidine, antibacterial and antifungal activities

## Abstract

The synthesis and antimicrobial evaluation of new 2-[6-methyl-4-(thietan-3-yloxy)pyrimidin-2-ylthio]acetohydrazide derivatives was investigated. According to the literature, there are a lot of antimicrobial agents among the pyrimidines and hydrazides, and therefore it seems promising to use 2-[6-methyl-4-(thietan-3-yloxy)pyrimidin-2-ylthio]acetohydrazide as a base object for synthesizing new biologically active substances. 2-[6-methyl-4-(thietan-3-yloxy)pyrimidin-2-ylthio]acetohydrazide was obtained by the hydrazinolysis of ethyl thioacetate, using a 3-fold molar excess of 85 % hydrazine hydrate in ethanol, at room temperature. Interaction of 2-[6-methyl-4-(thietan-3-yloxy)pyrimidin-2-ylthio]acetohydrazide with ketones during boiling in ethanol yielded N-ylidenehydrazides. The solid obtained by concentration was collected, and then purified by recrystallization. The new compounds were characterized by ^1^H, ^13^C NMR, IR spectroscopy and elemental analysis. The antibacterial and antifungal activities of the new compounds were analysed using agar diffusion and tenfold broth (pH 7.2 – 7.4) dilution methods, in comparison with the clinical used drugs, ceftriaxone and Pimafucin. The structure–activity studies showed that, depending on the nature of the hydrazide fragment, the newly synthesized compounds exhibited varying degrees of microbial inhibition. Within the same series the antimicrobial activity depends on the nature of the substituent attached to the benzene ring. The investigation of antibacterial screening data revealed that the compounds N′-[1-(4-aminophenyl)ethylidene]-2-[6-methyl-4-(thietan-3-yloxy)pyrimidin-2-ylthio]acetohydrazide, N′-[1-(4-hydroxyphenyl)ethylidene]-2-[6-methyl-4-(thietan-3-yloxy)pyrimidin-2-ylthio]acetohydrazide, N′-[1- (2,5-dihydroxyphenyl) ethylidene]-2-[6-methyl-4-(thietan-3-yloxy)-pyrimidin-2-ylthio]acetohydrazide were found to be more potent than the other synthesized analogues.

## Introduction

Infectious diseases are still a challenging global problem and a major public health threat, and this has led to the research and development of new antibiotics for multi-drug resistant microbial pathogens. Fungal and bacterial agents affect internal organs like the mucous membrane of the respiratory, gastrointestinal and urinary tracts, and cause different diseases [1,2]. Increasingly, there is a growing number of individuals with a weakened immune system who can get infected.

Over the last decade there has been considerable interest in the synthesis and pharmacological study of pyrimidine derivatives [3-7]. The pyrimidine fragment has been gaining special interest as it is part of many biological active compounds: vitamins, nucleotides and nucleosides. Our research group has published much research in the field of the synthesis of thietan derivatives [8,9] as they possess a broad spectrum of bioactivities: antimicrobial [10,11], hypotensive [12,13], broncholytic [14] and wound healing [15,16]. Compounds containing thietan and pyrimidine in their molecules are suitable candidates for further chemical modifications and may be pharmacologically active. We report the synthesis of new derivatives of 2-thiopyrimidine containing thietan ring, and investigate their antibacterial and antifungal properties.

## Experimental

### Materials and methods

The reagents used in the experiments were all commercially available without further purification. Each reaction was monitored by thin layer chromatography (TLC) on Sorbfil PTSX-AF-A-UV plates using ethyl acetate. Visualization on TLC was achieved by UV light or iodine indicator.

Melting points were recorded on PTP-M (Russia) melting point apparatus uncorrected. Infrared spectra were recorded in KBr pellets on an Infralum FT-02 (Russia) spectrophotometer. ^1^H and ^13^C NMR spectra were recorded on Bruker AMX-300 300 MHz and Bruker Avance III 500 MHz in CDCl_3_ and DMSO-d_6_ using tetramethylsilane as internal standard (chemical shifts were expressed as δ-values, *J* in hertz). CHNS elemental analyses were performed on a Hekatech Euro-EA (Germany) elemental analyzer.

**Figure 1.**
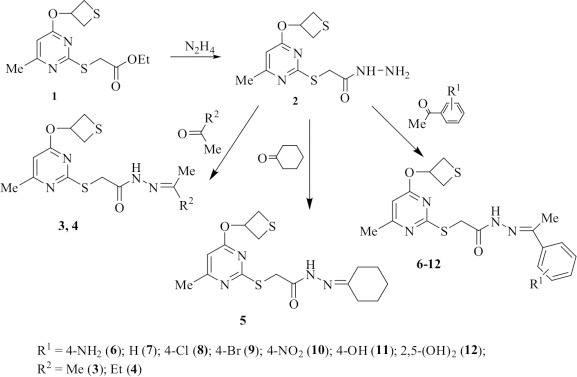
Synthetic route of the synthesized compounds **1**-**12**

Ethyl 2-[6-methyl-4-(tietan-3-yloxy)pyrimidine-2-ylthio]acetate (**1**) was synthesized by interaction of ethyl-2-(6-methyl-4-oxo-3,5-dihydropyrimidine-2-ilthio)acetate with 2-chloromethylthiirane [16] (Figure 1).

#### Ethyl 2-[6-methyl-4-(tietan-3-yloxy)pyrimidine-2-ylthio]acetate (compound **1**)

Light yellow crystalline powder. Recrystallization from mixture EtOH-water (3:1). Yield: 42 %; m.p. 61 °C. IR (KBr) in cm^−1^: 1630 (νС=О), 3280 (νN-H), 1530 (δN-H); 1572-1591 (С=С, С=N), 2960 (νC-H), 1443 (δC=C^6^-CH_3_), 1167, 1050 (C-O-C), 1750 (νCH_2_-COOR). ^1^H NMR (300 MHz, CDCl_3_, *δ*, ppm): 1.31 t (3Н, СН_3_, Et, *J* 7.1 Hz); 2.34 s (3H, 6-CH_3_); 3.38-3.48 m [2Н, S(CH)_2_]; 3.48-3.58 m [2Н, S(CH)_2_]; 3.82 s (2Н, SCH_2_CO); 4.22 q (2Н, ОCH_2_, Et, *J* 4,1 Hz); 5.55-5.57 m (1Н, OCH); 6.21 s (1H, 5-H). ^13^C NMR (125.5 MHz, CDCl_3_, *δ*, ppm): 168.52 (C^6^); 23.11 (^6^C-CH_3_); 101.91 (^5^CH); 166.95 (^4^C); 69.63 (OC^3^); 34.50 (SC^2,4^); 169.17 (^2^C); 32.51 (2-SCH_2_CO); 166.79 (2-SCH_2_CO), 60,61 (CH_2_CH_3_), 14,15 (CH_2_CH_3_). Found, %: C 47.96; H 5.39; N 9.43; S 21.35. C_12_H_16_N_2_O_3_S_2_. Calculated, %: C 47.98; H 5.37; N 9.33; S 21.35.

#### (*E,Z*)-2-[6-methyl-4-(thietan-3-yloxy)pyrimidin-2-ylthio]acetohydrazide (compound **2**)

Light yellow crystalline powder. 85% hydrazine hydrate (0.29 g, 0.00501 mol) solution was added to a solution of compound **1** (0.5 g, 0.00167 mol) in 3 ml of ethanol. The reaction mixture was mixed at room temperature for 4 hours. The product which precipitated was filtered, dried and recrystallized from i-PrOH. Yield: 0.32 g (67%); m.p. 119-120°C. IR (KBr) in cm^−1^: 1630 (νС=О), 3280 (νN-H), 1520 (δN-H); 1572-1591 (С=С, С=N), 2960 (νC-H), 1443 (δC=C^6^-CH_3_), 1167, 1050 (C-O-C). ^1^H NMR (300 MHz, C_6_D_6_, *δ*, ppm): 2.30 (3Н, s, ^6^C–CH_3_,), 3.42-3.49, (4H, m, S(CH_2_)_2_), 5.76-5.82 (1H, m, OCH), 6,50 (*Z*), 6,48 (*E*) (1H, s, ^5^C–H), 3,78 (*Z*), 4,18 (*E*) s (2Н, SCH_2_СО); 4,30 (*Z*), 4,54 (*E*) wid. s (2Н, NН_2_); 8,61 (*E*), 9,31 (*Z*) wid. s (1Н, NH). ^13^C NMR (125.5 MHz, CDCl_3_, *δ*, ppm): 168.52 (C^6^); 23.11 (^6^C-CH_3_); 101.91 (^5^CH); 166.95 (^4^C); 69.63 (OC^3^); 34.50 (SC^2,4^); 169.17 (^2^C); 32.51 (2-SCH_2_CO); 166.79 (2-SCH_2_CO). Found,%: C 41.95; H 4.93; N 19.57; S 22.39. C_10_H_14_N_4_O_2_S_2_. Calculated,%: C 41.94; H 4.93; N 19.57; S 22.39.

### General procedure for the synthesis of compound 3-12

Appropriate ketone was added to a solution of compound **2** in 5 ml of ethanol (Figure 1). The reaction mixture was boiled for 1 h. The solid obtained by concentration was collected and purified by recrystallization.

#### (*E,Z*)-2-[6-Methyl-4-(thietan-3-yloxy)pyrimidin-2-ylthio]-*N′*-(propan-2-ylidene)acetohydrazide (compound **3**)

White solid. Recrystallization from EtOH. Yield: 0.21 g (61%); m.p. 141-142 °C. IR (KBr) in cm^−1^: 1670 («amide I»), 1650 (C=O), 1588, 1572 (С=N, С=С), 1546 (δ NH, «amide II»), 1393 (δС-Н), 1164 (n_as_), 1045 (n_s_) (C-O-C). ^1^H NMR (300 MHz, C_6_D_6_, *δ*, ppm): 2.10 (3Н, s, ^6^C–CH_3_,), 3.12-3.19, 3.31-3.37 (4H, m, S(CH_2_)_2_), 5.62-5.63 (1H, m, OCH), 5.82 (1H, s, ^5^C–H), 4.44 (1H, s, SCH_2_CO), 9.94 (1H, wid s, CONH), 1.47 s (3Н, =С(СН_3_)); 1.65 s (3Н, =С(СН_3_)). ^1^H NMR (300 MHz, DMSO-*d_6_*, *δ*, ppm): 2.29 (*E*), 2.50 (*Z*) (3Н, s, ^6^C–CH_3_,), 3.35-3.49 (*Z*, *E*) (4H, m, S(CH_2_)_2_), 5.68-5.77 (*E, Z*) (1H, m, OCH), 6.45 (*E*), 6.48 (*Z*) (1H, s, ^5^C–H), 3.92 (*Z*), 4.26 (*E*) (1H, s, SCH_2_CO), 10.29 (*Z*), 10.39 (*E*) (1H, wid s, CONH), 1.90 (*E*), 1.91 (*Z*) s (3Н, =С(СН_3_)); 1.92 (*Z*), 1.94 (*E*) s (3Н, =С(СН_3_)). ^1^H NMR (300 MHz, CDCl_3_, *δ*, ppm): 2.35 (*Z*), 2.41 (*E*) (3Н, s, ^6^C–CH_3_,), 3.33-3.37, 3.41-3.45(*E*), 3.49-3.54 (*Z*) (4H, m, S(CH_2_)_2_), 5.78-5.86 (*E, Z*) (1H, m, OCH), 6.19 (*E*), 6.30 (*Z*) (1H, s, ^5^C–H), 3.87 (*Z*), 4.29 (*E*) (1H, s, SCH_2_CO), 8.56 (*Z*), 9.57 (*E*) (1H, wid s, CONH), 1.78 (*E*), 1.91 (*Z*) s (3Н, =С(СН_3_)); 2.05 (*Z*), 2.07 (*E*) s (3Н, =С(СН_3_)). ^13^C NMR (125,5 MHz, CDCl_3_, *δ*, ppm): 165.07 (*E*, *Z*) (C^6^); 25.41 (*E*); 25.48 (*Z*) (^6^C-CH_3_); 102.13 (*Z*); 103.30 (*E*) (^5^CH); 167.45 (*Z*); 167.65(*E*) (^4^C); 69.51 (*Z*); 70.29 (*E*) (OC^3^); 35.10 (*E*); 35.56 (*Z*) (SC^2,4^); 168.30 (*E*); 168.49 (*Z*) (^2^C); 33.35 (*Z*); 33.58 (*E*) (2-SCH_2_CO); 169.44 (*Z*); 170.02 (*E*) (2-SCH_2_CO); 16.10 (*Z*), 16.91 (*E*) (N=C(CH_3_)); 23.72 (*Z*), 23.93 (*E*) (N=C(CH_3_)); 150.21 (*Z*), 155.49 (*E*) (N=C). Found,%: C 47.83; H 5.56; N, 17.16; S 19.64. C_13_H_18_N_4_O_2_S_2_. Calculated,%: C 47.83; H 5.56; N, 17.16; S 19.64.

#### (*E,Z*)-*N*′-(Butan-2-ylidene)-2-[6-methyl-4-(thietan-3-yloxy)pyrimidin-2-yl-thio]acetohydrazide (compound **4**)

White solid. Recrystallization from EtOH. Yield: 0.29 g (81%); m.p. 131-132 °C. IR (KBr) in cm^−1^: 1675 («amide I»), 1645 (C=O), 1586, 1571 (С=N, С=С), 1546 (δ NH, «amide II»), 1393 (δС-Н), 1168 (n_as_), 1050 (n_s_) (C-O-C). ^1^H NMR (300 MHz, CDCl_3_, *δ*, ppm): 2.07 (*Z*), 2.12 (*E*) (3Н, s, ^6^C–CH_3_,), 3.42-3.45(*E*), 3.49-3.53 (*Z*) (4H, m, S(CH_2_)_2_), 5.73-5.86 (*E, Z*) (1H, m, OCH), 5.95 (*E*), 6.11 (*Z*) (1H, s, ^5^C–H), 4.01 (*Z*), 4.29 (*E*) (1H, s, SCH_2_CO), 9.57 (*Z*), 10.57 (*E*) (1H, wid s, CONH), 2.05 (*E*), 2.17 (*Z*) m (2Н, =ССН_2_); 1.92 (*Z*), 1.99 (*E*) s (3Н, =ССН_3_), 0.83 (*Z*), 1.08 (*E*) t (3Н, =ССН_2_CH_3_). ^13^C NMR (125.5 MHz, CDCl_3_, *δ*, ppm): 166.02 (*E*, *Z*) (C^6^); 23.74 (*E*); 23.88 (*Z*) (^6^C-CH_3_); 101.43 (*Z*); 102.30 (*E*) (^5^CH); 168.45 (*Z*); 168.65(*E*) (^4^C); 67.41 (*Z*); 68.39 (*E*) (OC^3^); 34.10 (*E*); 34.76 (*Z*) (SC^2,4^); 169.40 (*E*); 169.48 (*Z*) (^2^C); 35.36 (*Z*); 35.78 (*E*) (2-SCH_2_CO); 170.42 (*Z*); 171.01 (*E*) (2-SCH_2_CO); 13.20 (*Z*), 13.81 (*E*) (N=C(CH_3_)); 11.12 (*Z*), 12.11 (*E*) (CH_3_); 26.22 (*Z*), 26.92 (*E*) (N=C(CH_2_)); 158.11 (*Z*), 158.39 (*E*) (N=C). Found,%: C 49.39; H 5.92; N, 16.46; S 18.83. C_14_H_20_N_4_O_2_S_2_. Calculated,%: C 49.39; H 5.92; N, 16.46; S 18.83.

#### (*E,Z*)-*N*′-(Cyclohexylidene)-2-[6-methyl-4-(thietan-3-yloxy)pyrimidin-2-yl-thio]acetohydrazide (compound **5**)

White solid. Recrystallization from *n*-BuOH. Yield: 0.34 g (89%); m.p. 180-181 °C. IR (KBr) in cm^−1^: 1661 («amide I»), 1580, 1566 (С=N, С=С), 1546 (δ NH, «amide II»), 1449-1432 (n CH_2 cyclohexane_), 1164 (n_as_), 1047 (n_s_) (C-O-C), 768 (C-S). ^1^H NMR (300 MHz, CDCl_3_, *δ*, ppm): 2.06 (*Z*), 2.12 (*E*) (3Н, s, ^6^C–CH_3_,), 3.41-3.48(*E*), 3.37-3.45 (*Z*) (4H, m, S(CH_2_)_2_), 5.73-5.86 (*E, Z*) (1H, m, OCH), 5.95 (*E*), 6.11 (*Z*) (1H, s, ^5^C–H), 4.11 (*Z*), 4.21 (*E*) (1H, s, SCH_2_CO), 9.56 (*Z*), 10.51 (*E*) (1H, wid s, CONH), 2.34 t (4Н, =С(СН_2_)_2_); 1.65 t (6Н, (СН_2_)_3_). ^13^C NMR (125.5 MHz, CDCl_3_, *δ*, ppm): 166.14 (*E*, *Z*) (C^6^); 23.84 (*E*); 23.98 (*Z*) (^6^C-CH_3_); 101.23 (*Z*); 102.36 (*E*) (^5^CH); 168.25 (*Z*); 168.85(*E*) (^4^C); 66.34 (*Z*); 67.58 (*E*) (OC^3^); 34.12 (*E*); 34.78 (*Z*) (SC^2,4^); 169.30 (*E*); 169.58 (*Z*) (^2^C); 39.26 (*Z*); 40.71 (*E*) (2-SCH_2_CO); 171.12 (*Z*); 172.02 (*E*) (2-SCH_2_CO); 28.14 (*E, Z*) (N=C(CH_2_); 34.12 (*E, Z*) (N=C(CH_2_); 27.58 (*E, Z*) (2CH_2_); 25.8 (*E, Z*) (CH_2_); 167.21 (*Z*), 167.39 (*E*) (N=C). Found,%: C 52.44; H, 6.06; N, 15.29; S 17.51. C_16_H_22_N_4_O_2_S_2_. Calculated,%: C 52.44; H 6.05; N, 15.29; S 17.50.

#### (*E,Z*)-*N*′-[1-(4-Aminophenyl)ethylidene]-2-[6-methyl-4-(thietan-3-yloxy)pyrimidin-2-ylthio]acetohydrazide (compound **6**)

Pale yellow solid. Recrystallization from *n*-BuOH. Yield: 0.35 g (83%); m.p. 176-177 °C. . IR (KBr) in cm^−1^: 3470 (NH_2_), 3187-3299 (CH_arom_), 1667 («amide I»), 1588, 1574 (С=N, С=С), 1549 (δ NH, «amide II»), 1164 (n_as_), 1045 (n_s_) (C-O-C), 799 (C-S). ^1^H NMR (300 MHz, CDCl_3_, *δ*, ppm): 2.08 (*Z*), 2.23 (*E*) (3Н, s, ^6^C–CH_3_,), 3.28-3.34(*E*), 3.44-3.50 (*Z*) (4H, m, S(CH_2_)_2_), 5.79-5.90 (*E, Z*) (1H, m, OCH), 6.18 (*E*), 6.30 (*Z*) (1H, s, ^5^C–H), 3.87 (*Z*), 3.92 (*E*) (1H, s, SCH_2_CO), 8.69 (*Z*), 9.72 (*E*) (1H, wid s, CONH), 2.34 (*E*), 2.43 (*Z*) s (3H, N=C(CH_3_)); 6.18 (*E, Z*) s (2H, NH_2_); 6.67 (*E, Z*) d (2Н_arom_, *J* 8.4 Hz); 7.60 (*E, Z*) d (2Н_arom_, *J* 8.6 Hz). ^13^C NMR (125,5 MHz, CDCl_3_, *δ*, ppm): 165.97 (*E*, *Z*) (C^6^); 23.61 (*E*); 24.08 (*Z*) (^6^C-CH_3_); 102.29 (*E*); 103.54 (*Z*) (^5^CH); 167.46 (*E, Z*); (^4^C); 69.47 (*E*); 70.23 (*Z*) (OC^3^); 35.09 (*Z*); 35.49 (*E*) (SC^2,4^); 168.49 (*E, Z*) (^2^C); 33.36 (*E*); 33.80 (*Z*) (2-SCH_2_CO); 170.78 (*E, Z*) (2-SCH_2_CO); 12.95 (*E*), 13.69 (*Z*) (=C(CH_3_)); 126.15 (*E*), 126.63 (*Z*) (2СН_arom_); 129.51 (*E*), 129.69 (*Z*) (2СН_arom_); 137.45 (*E, Z*) (С_arom_); 148.29 (*E*), 149.05 (*Z*) (=C(CH_3_)). Found,%: C 53.58; H 5.25; N, 17.36; S 15.89. C_18_H_21_N_5_O_2_S_2_. Calculated,%: C 53.58; H 5.25; N, 17.36; S 15.89.

#### (*E,Z*)-2-[6-Methyl-4-(thietan-3-yloxy)pyrimidin-2-ylthio]-*N*′-(1-phenylethylidene)acetohydrazide (compound **7**)

White solid. Recrystallization from *n*-BuOH. Yield: 0.32 g (79%); m.p. 178-179 °C. IR (KBr) in cm^−1^: 3187-3299 (CH_arom_), 1667 («amide I»), 1588, 1574 (С=N, С=С), 1549 (δ NH, «amide II»), 1164 (n_as_), 1045 (n_s_) (C-O-C), 799 (C-S). ^1^H NMR (300 MHz, CDCl_3_, *δ*, ppm): 2.14 (*Z*), 2.30 (*E*) (3Н, s, ^6^C–CH_3_,), 3.28-3.31(*E*), 3.42-3.53 (*Z*) (4H, m, S(CH_2_)_2_), 5.77-5.87 (*E, Z*) (1H, m, OCH), 6.17 (*E*), 6.31 (*Z*) (1H, s, ^5^C–H), 3.93 (*Z*), 4.42 (*E*) (1H, s, SCH_2_CO), 9.14 (*E*), 9.87 (*Z*) (1H, wid s, CONH), 2.33 (*E*), 2.44 (*Z*) s (3H, N=C(CH_3_)); 7.38-7.42 (*E, Z*) m (3Н_arom_); 7.74-7.79 (*E, Z*) м (2Н_arom_). ^13^C NMR (125,5 MHz, CDCl_3_, *δ*, ppm): 165.98 (*E*, *Z*) (C^6^); 23.71 (*E*); 24.01 (*Z*) (^6^C-CH_3_); 102.19 (*E*); 103.34 (*Z*) (^5^CH); 167.46 (*E, Z*); (^4^C); 69.47 (*E*); 70.23 (*Z*) (OC^3^); 35.09 (*Z*); 35.49 (*E*) (SC^2,4^); 168.49 (*E, Z*) (^2^C); 33.36 (*E*); 33.80 (*Z*) (2-SCH_2_CO); 170.78 (*E, Z*) (2-SCH_2_CO); 12,95 (*E*), 13,69 (*Z*) (=C(CH_3_)); 126,15 (*E*), 126,63 (*Z*) (СН_arom_); 128,33 (*Z*), 128,47 (*E*) (СН_arom_); 129,51 (*E*), 129,69 (*Z*) (СН_arom_); 137,65 (*E, Z*) (С_arom_); 148,29 (*E*), 149,05 (*Z*) (=C(CH_3_)). Found,%: C 55.61; H 5.19; N, 14.42; S 16.50. C_18_H_20_N_4_O_2_S_2_. Calculated,%: C 55.65; H 5.19; N, 14.42; S 16.50.

#### (*E,Z*)-*N*′-[1-(4-Chlorophenyl)ethylidene]-2-[6-methyl-4-(thietan-3-yloxy)pyrimidin-2-ylthio]acetohydrazide (compound **8**)

White solid. Recrystallization from *i*-BuOH. Yield: 0.32 g (79%); m.p. 170-171 °C. IR (KBr) in cm^−1^: 1630 (nС=О), 3280 (nN-H), 1520 (dN-H); 1572-1591 (С=С, С=N), 2960 (nC-H), 1443 (dC=C^6^-CH_3_), 1167, 1050 (C-O-C), 1090 (C-Cl). ^1^H NMR (300 MHz, CDCl_3_, *δ*, ppm): 2.12 (*Z*), 2.18 (*E*) (3Н, s, ^6^C–CH_3_,), 3.26-3.31(*E*), 3.41-3.54 (*Z*) (4H, m, S(CH_2_)_2_), 5.77-5.85 (*E, Z*) (1H, m, OCH), 6.19 (*E*), 6.31 (*Z*) (1H, s, ^5^C–H), 3.93 (*Z*), 4.40 (*E*) (1H, s, SCH_2_CO), 9.02 (*E*), 9.90 (*Z*) (1H, wid s, CONH), 2.33 (*E*), 2.44 (*Z*) s (3H, N=C(CH_3_)); 7.37 (*E, Z*) d (2Н_arom_, *J* 8.4 Hz); 7.71 (*E, Z*) d (2Н_arom_, *J* 7.5 Hz). ^13^C NMR (125,5 MHz, CDCl_3_, *δ*, ppm): 163.78 (*E*, *Z*) (C^6^); 23.71 (*E*); 23.97 (*Z*) (^6^C-CH_3_); 102.22 (*E*); 103.37 (*Z*) (^5^CH); 167.45 (*E, Z*); (^4^C); 69.43 (*E*); 70.22 (*Z*) (OC^3^); 35.09 (*Z*); 35.49 (*E*) (SC^2,4^); 168.52 (*E, Z*) (^2^C); 33.30 (*E*); 33.78 (*Z*) (2-SCH_2_CO); 170.90 (*E, Z*) (2-SCH_2_CO); 12,87 (*E*), 13,48 (*Z*) (=C(CH_3_)); 123,87 (*E, Z*) (2СН_arom_); 131,49 (*Z*), 131,74 (*E*) (2СН_arom_); 136,86 (*E, Z*) (2С_arom_); 147,57 (*E, Z*) (=C(CH_3_)). Found,%: C 51.12; H 4.53; N 13.25; S 15.16. C_18_H_19_ClN_4_O_2_S_2_. Calculated,%: C 51.12; H 4.53; N 13.25; S 15.16.

#### (*E,Z*)-*N*′-[1-(4-Bromophenyl)ethylidene]-2-[6-methyl-4-(thietan-3-yloxy)pyrimidin-2-ylthio]acetohydrazide (compound **9**)

White solid. Recrystallization from *n*-BuOH. Yield: 0.40 g (81%); m.p. 178-179 °C. IR (KBr) in cm^−1^: 1630 (nС=О), 3280 (nN-H), 1520 (dN-H); 1572-1591 (С=С, С=N), 2960 (nC-H), 1443 (dC=C^6^-CH_3_), 1167, 1050 (C-O-C), 1050 (C-Br). ^1^H NMR (300 MHz, CDCl_3_, *δ*, ppm): 2.11 (*Z*), 2.28 (*E*) (3Н, s, ^6^C–CH_3_,), 3.26-3.30(*E*), 3.41-3.52 (*Z*) (4H, m, S(CH_2_)_2_), 5.75-5.87 (*E, Z*) (1H, m, OCH), 6.17 (*E*), 6.31 (*Z*) (1H, s, ^5^C–H), 3.92 (*Z*), 4.39 (*E*) (1H, s, SCH_2_CO), 9.30 (*E*), 9.91 (*Z*) (1H, wid s, CONH), 2.32 (*E*), 2.43 (*Z*) s (3H, N=C(CH_3_)); 6.91 (*E, Z*) d (2Н_arom_, *J* 8,7 Hz), 7.51 (*E, Z*) d (2Н_arom_, *J* 8,6 Hz). ^13^C NMR (125,5 MHz, CDCl_3_, *δ*, ppm): 163.78 (*E*, *Z*) (C^6^); 23.71 (*E*); 23.97 (*Z*) (^6^C-CH_3_); 102.22 (*E*); 103.37 (*Z*) (^5^CH); 167.45 (*E, Z*); (^4^C); 69.43 (*E*); 70.22 (*Z*) (OC^3^); 35.09 (*Z*); 35.49 (*E*) (SC^2,4^); 168.52 (*E, Z*) (^2^C); 33.30 (*E*); 33.78 (*Z*) (2-SCH_2_CO); 170.90 (*E, Z*) (2-SCH_2_CO); 12,87 (*E*), 13,48 (*Z*) (=C(CH_3_)); 123,87 (*E, Z*) (2СН_arom_); 131,49 (*Z*), 131,64 (*E*) (2СН_arom_); 136,56 (*E, Z*) (2С_arom_); 147,30 (*E, Z*) (=C(CH_3_)). Found,%: C 46.26; H 4.10; N, 11.93; S 13.72. C_18_H_19_BrN_4_O_2_S_2_. Calculated,%: C 46.26; H 4.10; N, 11.99; S 13.72.

#### (*E,Z*)-2-[6-Methyl-4-(thietan-3-yloxy)pyrimidin-2-ylthio]-*N*′-[1-(4-nitrophenyl)ethylidene]acetohydrazide (compound **10**)

Yellow solid. Recrystallization from *n*-BuOH. Yield: 0.35 g (76%); m.p. 169-170°C. IR (KBr) in cm^−1^: 1630 (nС=О), 3280 (nN-H), 1520 (dN-H); 1572-1591 (С=С, С=N), 2960 (nC-H), 1560 (n_as_), 1350 (n_s_) (NO), (1443 (dC=C^6^-CH_3_), 1167, 1050 (C-O-C). ^1^H NMR (300 MHz, CDCl_3_, *δ*, ppm): 2.39 (*Z*), 2.43 (*E*) (3Н, s, ^6^C–CH_3_,), 3.26-3.46(*E*), 3.51-3.71 (*Z*) (4H, m, S(CH_2_)_2_), 5.14-5.26 (*E, Z*) (1H, m, OCH), 6.11 (*E*), 6.26 (*Z*) (1H, s, ^5^C–H), 3.93 (*Z*), 4.05 (*E*) (1H, s, SCH_2_CO), 9.98 (*E*), 10.57 (*Z*) (1H, wid s, CONH), 2.39 (*E*), 2.43 (*Z*) s (3H, N=C(CH_3_)); 8.07 (*E, Z*) d (2Н_arom_, *J* 8,7 Hz), 8.34 (*E, Z*) d (2Н_arom_, *J* 8,6 Hz). ^1^H NMR (300 MHz, CDCl_3_, *δ*, ppm): 2.11 (*Z*), 2.28 (*E*) (3Н, s, ^6^C–CH_3_,), 3.26-3.30(*E*), 3.41-3.52 (*Z*) (4H, m, S(CH_2_)_2_), 5.75-5.87 (*E, Z*) (1H, m, OCH), 6.17 (*E*), 6.31 (*Z*) (1H, s, ^5^C–H), 3.92 (*Z*), 4.39 (*E*) (1H, s, SCH_2_CO), 9.30 (*E*), 9.91 (*Z*) (1H, wid s, CONH), 2.32 (*E*), 2.43 (*Z*) s (3H, N=C(CH_3_)); 6.91 (*E, Z*) d (2Н_arom_, *J* 8,7 Hz), 7.51 (*E, Z*) d (2Н_arom_, *J* 8,6 Hz). ^13^C NMR (125,5 MHz, CDCl_3_, *δ*, ppm): 163.78 (*E*, *Z*) (C^6^); 23.71 (*E*); 23.97 (*Z*) (^6^C-CH_3_); 102.22 (*E*); 103.37 (*Z*) (^5^CH); 167.45 (*E, Z*); (^4^C); 69.43 (*E*); 70.22 (*Z*) (OC^3^); 35.09 (*Z*); 35.49 (*E*) (SC^2,4^); 169.32 (*E, Z*) (^2^C); 40.89 (*E*); 41.20 (*Z*) (2-SCH_2_CO); 170.90 (*E, Z*) (2-SCH_2_CO); 12,87 (*E*), 16,48 (*Z*) (=C(CH_3_)); 127,77 (*E, Z*) (4СН_arom_), 150,26 (*E, Z*) (С_arom_); 143,60 (*E, Z*) (2С_arom_). Found,%: C 49.87; H 4.42; N, 16.16; S 14.79. C_18_H_19_N_5_O_4_S_2_. Calculated,%: C 49.87; H 4.42; N, 16.16; S 14.79.

#### (*E,Z*)-*N*′-[1-(4-hydroxyphenyl)ethylidene]-2-[6-methyl-4-(thietan-3-yloxy)pyrimidin-2-ylthio]acetohydrazide (compound **11**)

White solid. Recrystallization from EtOH. Yield: 0.33 g (79%); m.p. 188-189 °C. IR (KBr) in cm^−1^: 3564 (O-H), 3109, 3073 (С-Н_arom_), 1667 («amide I»), 1591, 1569 (С=N, С=С), 1541 (δ NH, «amide II»), 1513 (N=C), 1167 (n_as_), 1053 (n_s_) (C-O-C). ^1^H NMR (300 MHz, CDCl_3_, *δ*, ppm): 2.39 (*Z*), 2.43 (*E*) (3Н, s, ^6^C–CH_3_,), 3.26-3.47 (*E*), 3.52-3.71 (*Z*) (4H, m, S(CH_2_)_2_), 5.14-5.26 (*E, Z*) (1H, m, OCH), 6.11 (*E*), 6.26 (*Z*) (1H, s, ^5^C–H), 3.93 (*Z*), 4.05 (*E*) (1H, s, SCH_2_CO), 9.98 (*E*), 10.57 (*Z*) (1H, wid s, CONH), 1.69 (*E*), 1.81 (*Z*) s (3H, N=C(CH_3_)); 9.68 (*E, Z*) wid s (OН), 7.74 (*E, Z*) d (2Н_arom_, *J* 8,7 Hz), 6.82 (*E, Z*) d (2Н_arom_, *J* 8,6 Hz). ^13^C NMR (125,5 MHz, CDCl_3_, *δ*, ppm): 163.78 (*E*, *Z*) (C^6^); 23.71 (*E*); 23.97 (*Z*) (^6^C-CH_3_); 102.22 (*E*); 103.37 (*Z*) (^5^CH); 167.45 (*E, Z*); (^4^C); 69.43 (*E*); 70.22 (*Z*) (OC^3^); 35.09 (*Z*); 35.49 (*E*) (SC^2,4^); 168.52 (*E, Z*) (^2^C); 33.30 (*E*); 33.78 (*Z*) (2-SCH_2_CO); 170.90 (*E, Z*) (2-SCH_2_CO); 12,87 (*E*), 13,48 (*Z*) (=C(CH_3_)); 123,97 (*E, Z*) (2СН_arom_); 132,49 (*Z*), 133,64 (*E*) (2СН_arom_); 137,56 (*E, Z*) (2С_arom_); 147,30 (*E, Z*) (=C(CH_3_)). Found,%: C 53.44; H 4.98; N 13.85; S 15.85. C_18_H_20_N_4_O_3_S_2_. Calculated,%: C 53.45; H 4.98; N 13.85; S 15.85.

#### (*E,Z*)-*N*′-[1-(2,5-dihydroxyphenyl)ethylidene]-2-[6-methyl-4-(thietan-3-yloxy)-pyrimidin-2-ylthio]acetohydrazide (compound **12**)

White solid. Recrystallization from *i*-PrOH. Yield: 0.36 g (81%); m.p. 180-181 °C.. IR (KBr) in cm^−1^: 3254 (O-Н), 1667 («amide I»), 1591, 1572 (С=N, С=С), 1533 («amide II»), 1485 (N=C), 1167 (n_as_), 1044 (n_s_) (C-O-C). ^1^H NMR (300 MHz, CDCl_3_, *δ*, ppm): 2.07 (*Z*), 2.15 (*E*) (3Н, s, ^6^C–CH_3_,), 3.29-3.41 (*E*), 3.52-3.71 (*Z*) (4H, m, S(CH_2_)_2_), 5.14-5.26 (*E, Z*) (1H, m, OCH), 6.11 (*E*), 6.26 (*Z*) (1H, s, ^5^C–H), 3.93 (*Z*), 4.05 (*E*) (1H, s, SCH_2_CO), 9.98 (*E*), 10.57 (*Z*) (1H, wid s, CONH), 1.69 (*E*), 1.82 (*Z*) s (3H, N=C(CH_3_)); 13.18 (*E, Z*) wid s (OН), 9.46 wid s (OН),6.66 (*E, Z*) d (2Н_arom_, *J* 8,4 Hz), 6.75 (*E, Z*) d (2Н_arom_, *J* 8,6 Hz), 7.08 (*E, Z*) d (2Н_arom_, *J* 8,7 Hz). ^13^C NMR (125,5 MHz, CDCl_3_, *δ*, ppm): 163.78 (*E*, *Z*) (C^6^); 23.71 (*E*); 23.97 (*Z*) (^6^C-CH_3_); 102.22 (*E*); 103.37 (*Z*) (^5^CH); 167.45 (*E, Z*); (^4^C); 69.43 (*E*); 70.22 (*Z*) (OC^3^); 35.09 (*Z*); 35.49 (*E*) (SC^2,4^); 168.52 (*E, Z*) (^2^C); 33.30 (*E*); 33.78 (*Z*) (2-SCH_2_CO); 170.90 (*E, Z*) (2-SCH_2_CO); 12,87 (*E*), 13,48 (*Z*) (=C(CH_3_)); 116,37 (*E, Z*) (СН_arom_); 119.62 (*E, Z*) (СН_arom_); 120.42 (*E, Z*) (СН_arom_); 120.21 (*E, Z*) (С_arom_); 151.21 (*E, Z*) (С_arom_); 155.51 (*E, Z*) (С_arom_); 147,30 (*E, Z*) (=C(CH_3_)). Found,%: C 51.41; H 4.79; N, 13.32; S 15.25. C_18_H_20_N_4_O_4_S_2_. Calculated,%: C 51.41; H 4.79; N, 13.32; S 15.25.

### Biological studies

Antibacterial and antifungal activities of the novel 2-[6-methyl-4-(thietan-3-yloxy)pyrimidin-2-ylthio]acetohydrazide derivatives were analyzed using the agar diffusion and the tenfold broth (pH 7.2 – 7.4) dilution methods. Microbial strains of the department of Microbiology and Virology, Bashkir State Medical University deposited at L.A. Tarasevich State Institute of Standardization and Control of Biomedical Preparations, the Ministry of Health of the Russian Federation were used as test organisms: *E. coli*, *C. diversus, Ent. aerogenes*, *P. vulgaris*, *K. pneumoniae*, *Ps. aeruginosa*, *St. aureus*, *Str. pyogenes* and lower fungi *C. albicans*. Test compounds (10 mg) were weighed and dissolved in 1 ml DMSO. These solutions were diluted in beef extract broth to achieve a final concentration of 0.1 mg/ml (stock solution). The nutrient broth, which contained logarithmic serially twofold diluted amount of test compound inoculated with 2.0·10^6^ c.f.u/ml, was used. The cultures were incubated for 24 h at 37 °C and the growth was monitored visually. The lowest concentration (highest dilution) required to arrest the growth of bacteria was regarded as minimum inhibitory concentration (MIC).

## Results and Discussion

### Chemistry

This study was undertaken to synthesise acetohydrazide derivatives. Synthesis of the desired compound **2** was carried out as depicted in Figure 1. The structure of hydrazide **2** was confirmed by a complex of NMR spectroscopy methods.

**Figure 2.**
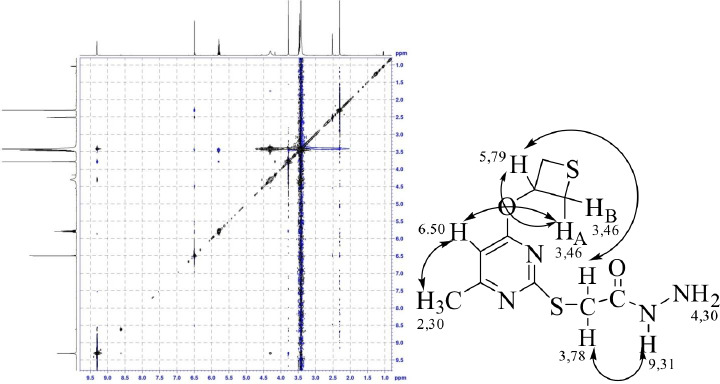
Two-dimensional ^1^H-^1^H ROESY spectrum (500 MHz, DMSO-d_6_) and structural schematic presentation of compound **2**

*E*,*Z*-isomerism was observed in acetohydrazide **2**, due to the inhibited rotation around the N-C hydrazide bond, as evidenced by the doubling of the proton signals of the thioacetohydrazide fragment. The spatial form of hydrazide **2** in DMSO-*d_6_* was established using two-dimensional NMR spectroscopy methods: NOESY and ROESY (Figure 2). According to the spectral data, the sterically more stable *Z*-conformer prevails, as evidenced by the presence of the nuclear Overhauser effect between the signal of the methylene protons of the acetamide group, and the signal of the proton bonded to the nitrogen atom of the hydrazide fragment, indicating that these protons are closer in space.

The signals of *Z-*conformer protons are registered in a stronger field in comparison with the same signals of the *E*-conformer. According to the ^1^H NMR spectra, the ratio of conformers is 19 (*Z*): 1(*E*).

These hydrazones might exist in the form of *E*,*Z*-isomers due to hindered rotation around the N-CO bond and *E*′,*Z*′-isomers around the C=N bond. The presence of two sets of chemical shifts in the ^1^H NMR spectra indicates that these compounds exist as a mixture of two stereoisomers.

Acetone hydrazone might exist only as *E*',Z'-isomers; and two sets of chemical shifts in the ^1^H NMR spectra can be caused only by hindered rotation around the N-CO bond.

Two sets of proton signals are recorded in the ^1^H NMR spectra of compound **3**, dissolved in polar solvents. When a solution of compound **3** in DMSO-*d_6_* is heated, coalescence occurs and the two sets of isomer signals merge into one. It confirms that the synthesized hydrazones in polar solvents exist as a mixture of two conformational *E*,*Z* isomers. Analysis of the ^1^Н NMR spectrum of hydrazone in DMSO-*d_6_* at 20.6° C showed that the signals of the protons of the СН_2_СО, NH groups - one of the methyl groups (СН_3_)_2_С=N of the *E* isomer - are shifted to a weaker field than the corresponding signals of the *Z*-isomer, which is consistent with the existing literature data. It is established that the sterically more stable *E*_N-CO_ isomer predominates, according to the analysis of the integral intensities of the ^1^H NMR spectrum signals of hydrazone **3**.

In the ^1^H NMR spectrum of hydrazone **3**, in non-polar solvents, i.e. benzene, one set of resonance signals was recorded, in which the values of the chemical shift indicate that hydrazone **3** in this case exists only in amide *E*-form.

### Antimicrobial and antifungal activity

Synthesized compounds were further screened for minimum inhibitory concentration against test organisms: *E. coli*, *C. diversus, Ent. aerogenes*, *P. vulgaris*, *K. pneumoniae*, *Ps. aeruginosa*, *St. aureus*, *Str. pyogenes* and lower fungi *C. albicans*. Minimum inhibitory concentration values are given in Table 1.

The investigation of antibacterial screening data revealed that the compounds **6**, **11** and **12** showed good inhibition towards all tested gram-positive and gram-negative bacteria, and lower fungi *C. albicans*. The derivative of 2-[6-methyl-4-(thietan-3-yloxy)pyrimidin-2-ylthio]acetohydrazide **7**, prepared in reaction with acetophenone, showed good antibacterial (MIC 0,05 – 0,5 μg/ml) and antifungal (MIC 0.05 μg/ml) activities, but it showed weak antimicrobial activity (MIC 0.5 μg/ml) against *Pseudomonas aeruginosa*. Compounds **8, 9** and **10** are less active against *St. aureus*, *P. vulgaris*. The results of antifungal testing revealed that compounds **1, 2, 4, 5, 9** and **10** are less active against *C. albicans*. All the other derivatives showed moderate activity against *C. albicans.*

The structure–activity studies showed that, depending on the nature of the hydrazide fragment, the newly synthesized compounds exhibited varying degrees of microbial inhibition. It was found that hydrazide showed better activity, as compared to the ester against *Str. pyogenes*, *P. vulgaris*, *K. pneumonia*, *Ent. aerogenes*, *E. cloacae*. The presence of alkyl fragments such as methyl and ethyl radicals decrease activity against *Str. pyogenes*, *P. vulgaris*. The presence of a cyclohexylidene substituent increases antimicrobial activities *E. coli*, *Ps. aeruginosa*, reduces antimicrobial activities against *St. aureus*, *Str. pyogenes*, *P. vulgaris*, *K. pneumonia*, *Ent. aerogenes*, *E. сloacae*. Within the same series the antimicrobial activity depends on the nature of the substituent attached to the benzene ring. Based on the data obtained it was possible to investigate some structure-activity relationships: OH, NH_2_ groups in the benzene fragment in the molecule led to an increase in antimicrobial and antifungal activities; compounds with halogen, NO_2_ groups in the benzene ring are the least active. The OH group attached to a benzene ring increased the inhibitory activity of compounds against *St. aureus. Str. pyogenes, P. vulgaris, Ps. aeruginosa*. Thus, the nature of the substituent on the benzene ring attached to 2-[6-methyl-4-(thietan-3-yloxy)pyrimidin-2-ylthio]acetohydrazide derivatives has a strong influence on the extent of antimicrobial activity.

## Conclusions

New 2-[6-methyl-4-(thietan-3-yloxy)pyrimidin-2-ylthio]acetohydrazide derivatives have been successfully obtained and tested as potential antimicrobial and antifungal agents. *N*-ylidenehydrazides were synthesized by the reaction of hydrazide with alkyl and aryl ketones, without the use of acid catalysers, with good yields of 61–89%. We have characterized a hydrazone-based molecular switch and studied the isomerization mechanism of this acetohydrazide system. Solvent and temperature induced switching between *E*,*Z*-isomers. The synthesized compounds **1-12** have been investigated for their *in vitro* antibacterial and antifungal activities. It was found that the compounds *N*′-[1-(4-Aminophenyl)ethylidene]-2-[6-methyl-4-(thietan-3-yloxy)pyrimidin-2-ylthio]acetohydrazide (compound **6**), *N*′-[1-(4-hydroxyphenyl)ethylidene]-2-[6-methyl-4-(thietan-3-yloxy)pyrimidin-2-ylthio]acetohydrazide (compound **11**), *N*′-[1-(2,5-dihydroxyphenyl)ethylidene]-2-[6-methyl-4-(thietan-3-yloxy)-pyrimidin-2-ylthio]acetohydrazide (compound **12**) obtained from the reaction of *p*-aminoacetophenone, p-hydroxyacetophenone and 2,5-dihydroxyacetophenone exhibit high antimicrobial and antifungal activity. Highest antifungal activity was observed in compounds **3**, **6**, **7**, **8**, **11** and **12**. Compounds that contain thietan and pyrimidine in their molecules are suitable candidates for further chemical modifications and are pharmacological active.

## Figures and Tables

**Table 1.** Antibacterial and antifungal activities of the newly prepared compounds

Compound	Minimum inhibitory concentrations (MIC), μg/ml								
	*St.* *aureus*	*Str.* *pyogenes*	*E.* *coli*	*P.* *vulgaris*	*K.* *pneumoniae*	*Ent.* *aerogenes*	*Ps.* *aeruginosa*	*E.* *сloacae*	*C.* *аlbicans*
**1**	0.5	0.5	0.5	0.5	0.5	0.5	0.5	0.5	0.5
**2**	0.5	0.05	0.5	0.05	0.05	0.05	0.5	0.05	0.5
**3**	50	5	0.5	5	0.05	0.05	0.05	0.05	0.05
**4**	5	0.5	0.5	0.5	0.05	0.05	5	0.05	0.5
**5**	50	5	0.05	5	5	0.5	0.05	5	0.5
**6**	0.05	0.05	0.05	0.05	0.05	0.05	0.05	0.5	0.05
**7**	0.5	0.5	0.5	0.5	0.05	0.05	0.5	0.05	0.05
**8**	50	0.5	0.5	5	0.5	0.5	0.5	0.5	0.05
**9**	50	5	5	5	0.5	5	5	0.5	5
**10**	50	5	0.5	5	5	0.5	0.05	5	5
**11**	0.05	0.05	0.05	0.05	0.05	0.05	0.05	0.05	0.05
**12**	0.05	0.05	0.5	0.05	0.05	0.05	0.05	0.05	0.05
ceftriaxone	0.5	0.5	0.5	0.5	0.05	0.05	0.05	0.5	0.05
Pimafucin	-	-	-	-	-	-	-	-	0.05
